# Phase II study of sunitinib in Japanese patients with unresectable or metastatic, well-differentiated pancreatic neuroendocrine tumor

**DOI:** 10.1007/s10637-012-9910-y

**Published:** 2013-10-01

**Authors:** Tetsuhide Ito, Takuji Okusaka, Toshirou Nishida, Kenji Yamao, Hisato Igarashi, Chigusa Morizane, Shunsuke Kondo, Nobumasa Mizuno, Kazuo Hara, Akira Sawaki, Satoshi Hashigaki, Nobuyuki Kimura, Mami Murakami, Emiko Ohki, Richard C. Chao, Masayuki Imamura

**Affiliations:** 1 Department of Medicine and Bioregulatory Science, Graduate School of Medical Science, Kyushu University, 3-1-1 Maidashi Higashi-ku, Fukuoka, Japan; 2 Hepatobiliary and Pancreatic Oncology Division, National Cancer Center Hospital, Tokyo, Japan; 3 Department of Surgery, Osaka Police Hospital, Osaka, Japan; 4 Department of Gastroenterology, Aichi Cancer Center Hospital, Nagoya, Japan; 5 Present Address: Nagoya Daini Red Cross Hospital, Nagoya, Japan; 6 Pfizer Japan Inc., Tokyo, Japan; 7 Pfizer Oncology, La Jolla, CA USA; 8 Kansai Electric Power Company Hospital, Osaka, Japan; 9 Present Address: Drug Delivery and Formulation Group, Medicinal Chemistry Platform, Ontario Institute for Cancer Research, Toronto, ON Canada

**Keywords:** Efficacy, Japanese, Pancreatic neuroendocrine tumor, Pharmacokinetics, Phase II, Sunitinib

## Abstract

*Background.* Pancreatic neuroendocrine tumors (NETs) are rare but are frequently diagnosed at advanced stages and require systemic therapy. *Patients and methods.* This multicenter, open-label, phase II study evaluated sunitinib in Japanese patients with well-differentiated pancreatic NET. Patients received sunitinib 37.5 mg/day on a continuous daily dosing (CDD) schedule. The primary endpoint was clinical benefit rate (CBR; percentage of complete responses [CRs] plus partial responses [PRs] plus stable disease [SD] ≥24 weeks). Secondary endpoints included objective response rate (ORR), tumor shrinkage, progression-free survival (PFS) probability, safety, pharmacokinetics, and biomarkers. *Results.* Twelve patients received treatment. The CBR was 75 % (95 % confidence interval [CI], 43–94) and included 6 patients with a PR and 3 with SD. The ORR was 50 % (95 % CI, 21–79). PFS probability was 91 % (95 % CI, 54–99) at 6 months and 71 % (95 % CI, 34–90) at 12 months. Commonly reported treatment-emergent (all-causality), any-grade adverse events included diarrhea (*n* = 10), hand–foot syndrome and hypertension (both *n* = 8), fatigue and headache (both *n* = 7), and neutropenia (*n* = 6). No deaths on study were reported; one death due to disease progression occurred >28 days after end of treatment. Sunitinib on a CDD schedule resulted in sustained drug concentrations without accumulation across cycles. Tumor responses in all 12 patients did not appear to correlate with decreases in chromogranin A levels. *Conclusions.* Sunitinib 37.5 mg/day on a CDD schedule demonstrated antitumor activity in Japanese patients with unresectable, well-differentiated pancreatic NET. Commonly reported adverse events were consistent with the known safety profile of sunitinib.

**Electronic supplementary material:**

The online version of this article (doi:10.1007/s10637-012-9910-y) contains supplementary material, which is available to authorized users.

## Introduction

Pancreatic neuroendocrine tumors (NETs) are rare malignancies with a prevalence of 2.23 per 100,000 population in Japan [1]. The incidence rate of pancreatic NET per year in Japan (1.01 per 100,000 population) appears higher than in Western countries (0.32/100,000 in the overall US population and 0.25 in Asian Americans [2]). Surgery, if feasible, is the optimal treatment approach [3]. However, the majority of patients present with unresectable disease. When the current study was initiated, treatment options available for symptomatic patients with unresectable disease included somatostatin analogs (e.g. octreotide, alone or in combination with interferon-alpha) and the alkylating agent streptozocin (alone or in combination with doxorubicin), both of which have limited efficacy in patients with advanced disease [4–6]. Subsequently, targeted anti-cancer agents have been shown to improve progression-free survival (PFS) compared with placebo in phase III studies that included primarily Caucasian patients with advanced pancreatic NET [7–9].

Vascular endothelial growth factor (VEGF) and platelet-derived growth factor (PDGF) are key drivers of angiogenesis in pancreatic NETs [10, 11]. Sunitinib malate (SUTENT®), an oral multitargeted inhibitor of numerous receptor tyrosine kinases including VEGF receptors and PDGF receptors [12–14], has been shown to delay tumor growth in a RIP1-Tag2 transgenic mouse model of pancreatic islet-cell tumors [15, 16]. In a phase II trial, sunitinib demonstrated antitumor activity in patients with pancreatic NET [17], and in a subsequent phase III trial, oral sunitinib 37.5 mg/day on a continuous daily dosing (CDD) schedule prolonged median PFS relative to placebo in Caucasian and Asian patients with locally advanced and/or metastatic, well-differentiated pancreatic NET [7]. Sunitinib was also associated with a greater objective tumor response rate than placebo. In an updated analysis, median overall survival (OS) favored sunitinib (hazard ratio [HR] 0.71, 95 % confidence interval [CI]: 0.47–1.09; *P* = 0.11), despite crossover to sunitinib for most of the patients randomized to placebo, although statistical significance was not reached [18]. On the basis of these findings, sunitinib has been approved multinationally for the treatment of patients with unresectable or metastatic, well-differentiated pancreatic NET with disease progression.

We carried out a phase II, open-label, multicenter trial (NCT01121562) to evaluate the clinical benefit rate (CBR) of sunitinib in Japanese patients with pancreatic NET. The sunitinib dose investigated was 37.5 mg/day on the CDD schedule, which was the same regimen used in a Western phase III study [7]. Secondary objectives were to assess objective response rate (ORR) and PFS, to evaluate safety and tolerability, and to determine the pharmacokinetic (PK) profile of sunitinib in this patient population.

## Patients and methods

### Study population

Japanese patients ≥20 years old with histologically or cytologically proven, well-differentiated pancreatic NET (according to the World Health Organization 2004 classification [19]) and progressive unresectable advanced or metastatic disease were eligible to participate. Inclusion criteria comprised documented evidence of disease progression within 12 months of study start (by computed tomography [CT] or magnetic resonance imaging [MRI]), and disease not amenable to surgery, radiation, or combined modality therapy with curative intent. At least one measurable target lesion according to Response Evaluation Criteria in Solid Tumors (RECIST) version 1.0 [20] was required, along with adequate organ function, an Eastern Cooperative Oncology Group performance status (ECOG PS) of 0 or 1, and a life expectancy of at least 3 months. Patients were excluded if they had poorly differentiated tumors, prior treatment with any tyrosine kinase or anti-VEGF angiogenic inhibitors, brain metastases, cardiovascular disease ≤12 months prior to study start, uncontrolled hypertension, an uncontrolled thyroid abnormality, ongoing cardiac dysrhythmias with medical intervention or a prolonged QT interval corrected for heart rate (QTc), symptomatic brain metastases, or a left ventricular ejection fraction of ≤50 %.

### Study design and treatment

In this multicenter, open-label, phase II study, all patients received oral sunitinib37.5 mg/day on a CDD schedule, and each treatment cycle lasted 28 days. Patients were monitored for toxicity, and dose reductions to 25 mg/day were permitted based on individual tolerability. The sunitinib dose could also be increased to 50 mg/day (if no response was observed in the first 8 weeks and if individual tolerability permitted). The primary endpoint was CBR, defined as the proportion of patients with a confirmed complete response (CR) or partial response (PR) or stable disease (SD) for ≥24 weeks. CBR was selected as the primary endpoint because maintaining prolonged SD over about half of a year (24 weeks) was deemed beneficial and clinically meaningful for patients with pancreatic NET, based on the median PFS of 5.5 months reported for placebo treatment in a previous global, pivotal, phase III study [7]. Secondary efficacy endpoints included ORR, defined as the proportion of patients with a confirmed CR or PR; tumor shrinkage, defined as the percentage change from baseline in the sum of the longest diameter of target lesions; PFS; safety; and PK.

The study was conducted in accordance with the International Conference on Harmonization Good Clinical Practice guidelines, the Declaration of Helsinki, and applicable local regulatory requirements and laws. Approval from the institutional review board or independent ethics committee of each participating center was required, and written informed consent was obtained from all patients before screening.

### Study assessments

Investigator-assessed tumor imaging by CT, spiral CT, or MRI was performed at screening and weeks 5 and 9, and then at 8-week intervals during the study. Additional scans were performed when disease progression was suspected or to confirm a CR or PR based on RECIST. Safety was assessed at regular intervals by physical examination and analysis of adverse events (AEs), laboratory abnormalities (hematology and blood chemistry), vital signs, 12-lead electrocardiograms (ECGs), and ECOG PS. AEs were graded using National Cancer Institute Common Terminology Criteria for Adverse Events, version 4.0. QTc intervals were determined using 12-lead ECGs in triplicate at baseline, on day 1 of cycles 2 and 3, every 8 weeks thereafter, and as clinically indicated.

Blood samples were collected before dosing on day 15 (±1) of cycle 1 and on day 1 of cycles 2–4 to evaluate trough concentrations (C_trough_) of sunitinib and its active metabolite SU12662 using a validated high- performance liquid chromatography − tandem mass spectrometry method (Bioanalytical Systems Inc., West Lafayette, Indiana, USA). An exploratory analysis investigated potential differences in steady-state C_trough_ values of sunitinib, SU12662, and total drug (sunitinib plus SU12662) in Japanese versus non-Japanese patient populations and in patients with different tumor types. C_trough_ from this Japanese study, a Western pancreatic NET trial [15], and from studies of Japanese patients with GIST or RCC [21, 22] were dose-corrected to 37.5 mg and compared.

Blood samples were obtained at screening, week 5, and week 9, and then every 8 weeks to assess chromogranin A (CgA) levels (all patients) and hormone levels (patients with functional tumors only). Patients were required to fast for ≥10 h prior to each scheduled visit. In an exploratory analysis of CgA levels, a biochemical response was defined as a ≥50 % decrease in CgA levels among patients with elevated CgA levels at baseline.

### Statistical methods

As pancreatic NETs are rare, a target sample size of at least 10 patients was determined based on feasibility of study conduct rather than statistical requirements. All enrolled patients who received at least one dose of study treatment were included in the efficacy and safety analyses.

Descriptive statistics were used to summarize patient characteristics, treatment administration/compliance, safety parameters, and PK variables. For the analysis of the primary endpoint, the CBR and its exact 95 % CI were calculated. For the analysis of the secondary endpoints, the ORR and its exact 95 % CI, and the percentage change from baseline in the sum of the longest diameter of target lesions were calculated. Time-to-event endpoints (PFS and OS) were summarized using Kaplan–Meier methods.

## Results

### Patients and treatment

Between July and December 2010, 12 patients (8 male, 4 female) were enrolled in the study at four centers in Japan. All patients received treatment and were analyzed for efficacy and safety. At data cut-off (March 2012), treatment was ongoing in 5 patients with a PR (*n* = 4) or SD (*n* = 1), and 7 patients had withdrawn from the trial. Study withdrawals were due to tumor progression or recurrence (*n* = 3), withdrawal of consent (*n* = 1), treatment interruption >4 weeks due to a serious adverse event (SAE; grade 4 enterocolitis; *n* = 1), and SAEs (grade 4 convulsion plus grade 4 loss of consciousness; *n* = 1). Demographic and baseline disease characteristics are presented in Table 1. All of the patients had well-differentiated pancreatic NETs, of which 10 were classified as nonfunctional and 2 as functional (both gastrinomas). Six patients had received prior octreotide treatment and continued octreotide therapy during the study.

**Table 1:** Patient characteristics at baseline

Patient characteristic	Sunitinib (*N* = 12)
Age, years	
Median	54
Range	34–79
Sex, *n* (%)	
Male	8 (67)
Female	4 (33)
ECOG performance status, *n* (%)	
0	11 (92)
1	1 (8)
Time since diagnosis, years	
Median	3
Range	0.2–9.0
Tumor functionality, *n* (%)	
Nonfunctioning	10 (83)
Functioning	2 (17)
Gastrinoma	2 (17)
Number of involved disease sites per patient, *n* (%)	
1 site	4 (33)
2 sites	5 (42)
3 sites	2 (17)
4 sites	1 (8)
Presence of distant metastases, *n* (%)	
Any, including hepatic	12 (100)
Extrahepatic	3 (25)
Involved disease sites, *n* (%)	
Liver	12 (100)
Lymph node	4 (33)
Pancreas	4 (33)
Lung	2 (17)
Bone	1 (8)
Peritoneum	1 (8)
Prior surgery, *n* (%)	
Yes	9 (75)
No	3 (25)
Prior radiation therapy, *n* (%)	
Yes	1 (8)
No	11 (92)
Number of prior systemic chemotherapy regimens, *n* (%)	
0	6 (50)
1	4 (33)
2	0
≥3	2 (17)

*ECOG* Eastern cooperative oncology group

The median relative sunitinib dose intensity was 51 % (range, 26–94); the median number of treatment cycles started was 16 (range, 3–21; Table 2). The sunitinib dose was interrupted in 11 patients and reduced in 8 patients. The most frequently reported cause of dosing interruptions or reductions was AEs.

**Table 2:** Sunitinib treatment

	Sunitinib (*N* = 12)
Treatment cycles started, median (range)	16 (3–21)
Months on treatment, median (range)	10 (0.7–18)
Months on study, median (range)	14 (0.7–19)
No. of patients with ≥1 dosing interruption, *n* (%)	11 (92)
No. of patients with ≥1 dose reduction, *n* (%)	8 (67)
Relative dose intensity, median (range), %	51 (26–94)

### Efficacy

**Fig. 1:**
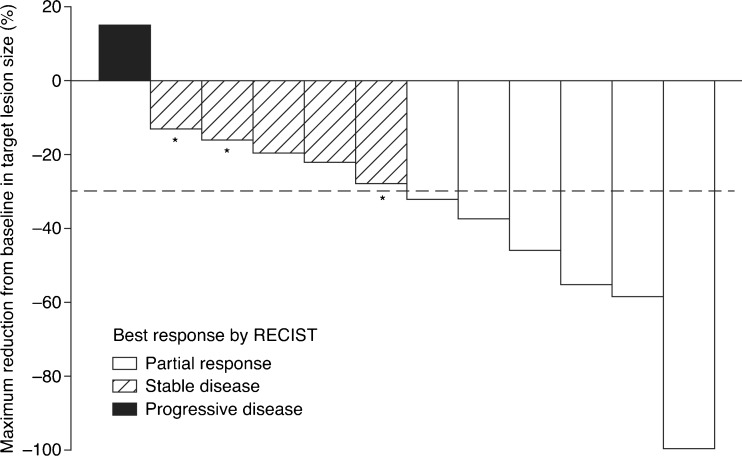
Maximum percentage reduction from baseline in target lesion size by patient (*N* = 12). Although one patient had a maximum percentage change in target tumor size from baseline of −100 %, non-target lesions remained and therefore this was not classified as a complete response. *Asterisk* stable disease of ≥24 weeks in duration; *RECIST* Response Evaluation Criteria in Solid Tumors

**Fig. 2:**
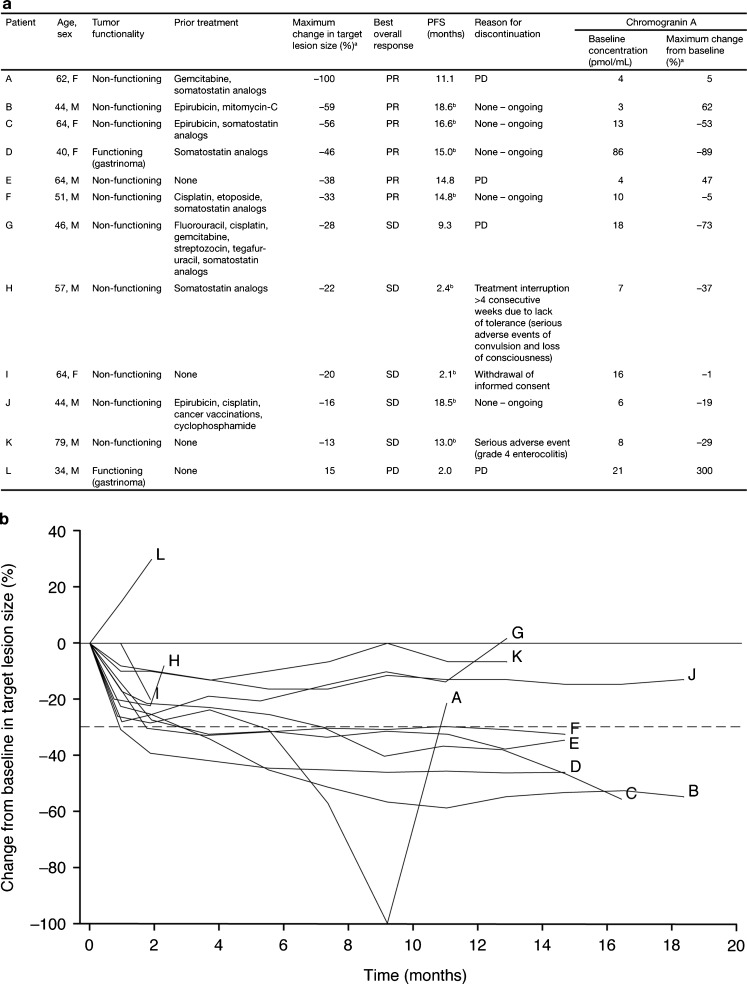
Individual patient profiles and response to treatment (*N* = 12). **a** Summary of patient profiles and changes in tumor-size and chromogranin A levels. **b** Percentage change from baseline in target lesion size over time in individual patients. ^a^ Maximum % change = [(minimum value after dosing – baseline)/baseline] x 100; ^b^ Based on censored data; *F* female; *M* male; *PD* progressive disease; *PFS* progression-free survival; *PR* partial response; *SD* stable disease

Based on investigator assessments, 6 of the 12 patients experienced a PR, and none had a CR (Fig. 1). SD ≥24 weeks was observed in 3 patients, and the CBR was 75 % (95 % CI, 43–94). In total, 5/6 patients with prior or concurrent octreotide treatment and 4/6 patients who did not receive octreotide met the criteria for experiencing clinical benefit. The overall ORR was 50 % (95 % CI, 21–79; Fig. 1). One patient showed a 100 % decrease in target lesion size. One PR occurred in a patient with gastrinoma and was accompanied by a 93 % decrease in plasma gastrin levels (Patient D; Data Supplement Table S1; see also below). In 11/12 patients, some degree of tumor shrinkage was observed by the first assessment 1 month after initiation of sunitinib treatment (Fig. 2). At the time of data cut-off, ongoing patients had been observed for at least 16.1 months, and the median duration of treatment with sunitinib was 9.8 months (range, 0.7–18.1). Although 4/12 patients had discontinued treatment with sunitinib due to reasons other than progressive disease (PD), median PFS had not yet been reached. Six-month and 12-month PFS probabilities were 91 % (95 % CI, 54–99) and 71 % (95 % CI, 34–90), respectively. Median OS had not yet been reached. One death occurred (due to progression of primary disease) during survival follow-up, more than 28 days after the end of treatment.

### Safety

As of March 2012, the most common treatment-emergent (all-causality) AEs of any grade were diarrhea (*n* = 10; 83 %), hand–foot syndrome and hypertension (both *n* = 8; 67 %); fatigue and headache (both *n* = 7; 58 %), and neutropenia (*n* = 6; 50 %; Table 3). Grade 3 AEs reported in at least 2 patients were neutropenia (*n* = 6; 50 %) and leukopenia (*n* = 2; 17 %). Four patients (33 %) experienced grade 4 AEs, all of which were judged to be related to treatment (herpes encephalitis, convulsion, and loss of consciousness [*n* = 1], increased lipase [*n* = 2], and enterocolitis [*n* = 1]). No deaths related to sunitinib treatment were reported on study or within 28 days of the end of treatment. One death due to disease progression occurred >28 days after end of treatment.

**Table 3:** Treatment-emergent (all-causality) adverse events (AEs) reported in ≥25 % of patients, according to the National Cancer Institute Common Terminology Criteria for Adverse Events version 4.0

	Maximum grade (G), *n* (%)			
AE	G1	G2	G3	Total^a^
Any AE^b^	0	0	8	12 (100)
Diarrhea	4 (33)	5 (42)	1 (8)	10 (83)
Hand–foot syndrome	1 (8)	7 (58)	0	8 (67)
Hypertension	1 (8)	7 (58)	0	8 (67)
Fatigue	1 (8)	6 (50)	0	7 (58)
Headache	4 (33)	3 (25)	0	7 (58)
Neutropenia	0	0	6 (50)	6 (50)
Dysgeusia	5 (42)	0	0	5 (42)
Nasopharyngitis	4 (33)	1 (8)	0	5 (42)
Nausea	4 (33)	1 (8)	0	5 (42)
Pyrexia	2 (17)	2 (17)	1 (8)	5 (42)
Vomiting	5 (42)	0	0	5 (42)
Decreased appetite	4 (33)	0	0	4 (33)
Edema	3 (25)	1 (8)	0	4 (33)
Hypothyroidism	0	4 (33)	0	4 (33)
Leukopenia	0	1 (8)	2 (17)	3 (25)
Mucosal inflammation	2 (17)	1 (8)	0	3 (25)
Muscle spasms	3 (25)	0	0	3 (25)
Prolonged electrocardiogram QT	1 (8)	1 (8)	1 (8)	3 (25)
Thrombocytopenia	1 (8)	1 (8)	1 (8)	3 (25)

^a^Grade 4 AEs were observed in 4 patients: convulsion, loss of consciousness, and herpes encephalitis (*n* = 1), increased lipase (*n* = 2), and enterocolitis (*n* = 1); no grade 5 AEs were reported

^b^Patients were counted once, with only the highest grade AE listed

Three patients (25 %) experienced SAEs, all of which resolved. In two cases, the SAEs were assessed as treatment-related. One patient (patient H; Fig. 2) had a grade 4 convulsion and grade 4 loss of consciousness that were reported to be likely due to herpes encephalitis. These SAEs resulted in a sunitinib dose interruption exceeding 4 weeks that led to study discontinuation, as specified in the protocol. Another patient (patient K; Fig. 2) experienced an SAE of grade 4 enterocolitis and temporarily discontinued therapy due to this SAE.

### Pharmacokinetics

Steady-state concentrations of sunitinib, SU12662, and total drug (sunitinib plus SU12662) were reached by day 15 of cycle 1. Subsequent sampling on day 1 of cycles 2–4 showed the concentrations to be sustained following CDD with sunitinib without disproportionate accumulation across cycles (data not shown). Mean dose-corrected (reference dose: 37.5 mg) C_trough_ values were within the ranges of 41.7–53.9 ng/mL for sunitinib, 19.6–25.7 ng/mL for SU12662, and 62.9–77.5 ng/mL for total drug.

**Fig. 3:**
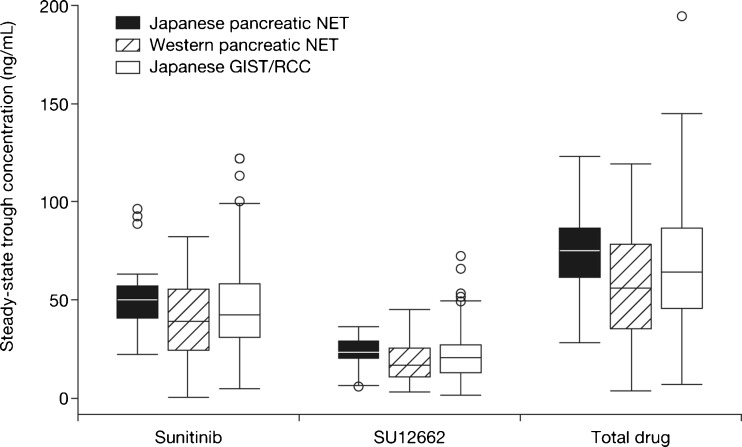
Trough concentrations of sunitinib, active metabolite SU12662, and total drug (sunitinib plus SU12662) in Japanese patients with pancreatic neuroendocrine tumor (NET; *n* = 11; present study) or gastrointestinal stromal tumor (GIST; *n* = 30) [21] and renal cell carcinoma (RCC; *n* = 38; pooled data) [25], and in predominantly Western patients with pancreatic NET; *n* = 57 [17]. The sunitinib dose in each study was dose-corrected to 37.5 mg. The *upper* and *lower box boundaries* denote the 75th and 25th percentiles, respectively, with the median shown as a line within the box. *Whiskers* indicate minimum and maximum values. Outlying values are denoted as *circles*

We explored potential differences in steady-state C_trough_ values of sunitinib and SU12662 in Japanese versus non-Japanese patient populations and in patients with different tumor types. Dose-corrected steady-state C_trough_ levels from this Japanese study were compared with findings from a Western pancreatic NET population [17], and from studies of Japanese patients with gastrointestinal stromal tumor (GIST) or renal cell carcinoma (RCC) [21, 22]. Steady-state C_trough_ levels of sunitinib, SU12662, or total drug were not significantly different between Japanese and primarily Western patients with pancreatic NET, or between patients with pancreatic NET, GIST, and RCC tumor types (Fig. 3).

### Biomarkers

#### Chromogranin A

Plasma CgA levels were measured in all 12 patients (Fig. 2). At baseline, the median CgA concentration was 9 pmol/mL (range, 3–86 pmol/mL). Six patients had above-median CgA levels at baseline, 3 of whom had a maximum percentage decrease in CgA concentrations of at least −50 %. Among these 3 patients, 2 had a PR and 1 had a best overall response of SD. In the patient with SD, the maximum percentage change in tumor size from baseline was −28 %. Among the 6 patients with below-median CgA levels, 3 experienced a PR and 3 had a best overall response of SD. Tumor responses in all 12 patients did not appear to correlate with the maximum percentage decrease in CgA levels.

#### Gastrin

Plasma gastrin levels were assessed in the 2 patients with gastrinomas: a 40-year-old female (patient D) and a 34-year-old male (patient L; Data Supplement Table S1). In addition, the relationship between hormonal levels, tumor size, and objective tumor response (based on investigator assessment) was examined in an exploratory analysis. In the male patient, neither gastrin levels nor tumor size decreased after treatment with sunitinib. The best objective response was PD, and the patient discontinued the study at day 79 due to lack of efficacy. In the female patient with gastrinoma, decreases in both gastrin levels (−85 % to −93 %) and tumor size (−31 % to −45 %) were observed during treatment. This patient had a PR on cycle 2 day 1 that was maintained through cycle 7 day 1.

## Discussion

This is the first report of sunitinib safety, PK profiles, PFS, and antitumor activity in Japanese patients with unresectable, advanced/metastatic well-differentiated pancreatic NET. While the data are limited by the small sample size, antitumor activity was observed in this population, with a CBR of 75 % and an ORR of 50 %. The ORR was encouraging and higher than the 9 % ORR reported for sunitinib in a randomized, phase III trial in a predominantly non-Asian population [7]. In the present study, 11 patients had decreases in target lesion measurements and achieved a best response of a PR or SD. Analysis of the percentage change in target lesion size over time showed a trend in tumor shrinkage from the first assessment 1 month after initiation of treatment with sunitinib. As of March 2012, ongoing patients had been observed for at least 16.1 months, and median PFS had not been reached. The probability of being alive and progression-free at 6 months was 91 % (95 % CI, 54–99) and at 12 months was 71 % (95 % CI, 34–90). In the randomized sunitinib phase III trial, median PFS was 11.4 months [7]. These data suggest that PFS in Japanese patients receiving sunitinib in our study may be equivalent to or greater than that observed in the randomized phase III trial.

The potential effect of octreotide therapy was difficult to evaluate in this study due to the small sample size. The CBR was similar among patients with (*n* = 5/6) or without (*n* = 4/6) octreotide treatment. In an exploratory subpopulation analysis reported in the randomized sunitinib phase III study, the efficacy of sunitinib appeared similar in patients who did and did not receive somatostatin analogues (PFS hazard ratios of 0.43 and 0.41, respectively), suggesting that the efficacy of sunitinib was not affected by somatostatin analogue treatment [7].

AEs reported during sunitinib treatment were manageable with palliative care measures, such as dosing interruption. The AEs observed in this study were similar to those reported in a phase III study in primarily Western patients with pancreatic NET [7], and frequently observed AEs were comparable to those reported in patients with GIST or RCC [21, 22].

In the current study, the median number of treatment cycles started was 16. All patients had grade 3/4 AEs and at least one sunitinib dosing interruption. However, the therapeutic effect of sunitinib did not appear to be reduced by temporary dosing interruptions due to AEs. Neutropenia, the most common grade 3/4 AE, was also the most frequently reported grade 3/4 toxicity in the predominantly Western sunitinib phase III pancreatic NET study [7], although the frequency of this event was markedly higher in our study with Japanese patients (42 % vs. 12 %). Increased rates of grade 3/4 neutropenia have also been observed in sunitinib-treated Japanese patients with GIST (37 % vs. 10 %) or RCC (53 % vs. 18 %), compared with predominantly Western populations [21–24]. The frequency of grade 3/4 thrombocytopenia similarly appeared to be higher in sunitinib-treated Japanese vs. Western patients (GIST: 20 % vs. 4 %; RCC: 55 % vs. 9 %; pancreatic NET: 17 % vs. 4 %) [21–24]. It should be noted that the sunitinib GIST and RCC trials used a different dosing schedule (sunitinib 50 mg/day, for 4 weeks on therapy, followed by 2 weeks off) than our study and the phase III, pancreatic NET trial.

It is not clear why rates of grade 3/4 hematologic AEs appear to be higher in Japanese versus Western patients who receive sunitinib. Analysis of PK parameters has shown that the area under the concentration–time values of sunitinib and SU12662 are similar in Japanese and Caucasian patients with RCC [22]. In addition, when steady-state C_trough_ values from this Japanese study were compared with those from a Western pancreatic NET population [17] and with C_trough_ values from Japanese patients with GIST or RCC [21, 22], there were no significant differences in the dose-corrected C_trough_ levels of sunitinib, SU12662, or total drug between Japanese and primarily Western patients with pancreatic NET, or among patients with pancreatic NET, GIST, or RCC. In the absence of racial or ethnic differences in the PK of sunitinib, Uemura et al. [25] suggested that the elevated rates of grade 3/4 hematologic AEs in Japanese patients may be due to differences in the expression levels and activity of sunitinib-sensitive kinases involved in the regulation of hematopoiesis.

Everolimus, an inhibitor of the mammalian target of rapamycin, is approved for the treatment of pancreatic NET in Japan, and like sunitinib is commonly associated with skin and gastrointestinal disorders [9]. Additional AEs related to sunitinib treatment include hematotoxicity, cardiovascular disorders and constitutional symptoms [26], while pneumonitis and infections are associated with everolimus therapy [27]. These different safety profiles reflect each compound’s distinct mode of action. No racial differences between Japanese and Western patients have been reported for the safety profile of either drug, based on the current study and a subgroup analysis of Japanese patients in the RADIANT-3 everolimus trial [9].

Treatment-emergent changes in CgA levels may provide a means to select patients with pancreatic NET likely to benefit from molecular targeted therapy [28]. However, in this study tumor responses in all 12 patients did not appear to correlate with the maximum percentage decrease in CgA levels, possibly because of small patient numbers with elevated CgA concentrations at baseline. Patient D had the highest baseline CgA levels in the study (86 pmol/mL), and decreased CgA concentrations (−89 %) were subsequently observed in combination with a PR. An increase in CgA levels (300 %) occurred during the study in 1 patient (Patient L) who experienced PD. A potential correlation between changes in CgA levels and clinical benefit was considered in these 2 patients. In patients with elevated baseline CgA concentrations, CgA appeared to be a useful marker in patients with pancreatic NET as reported previously [29].

The use of sunitinib marks a new phase in the development of a more targeted approach to the treatment of advanced-stage pancreatic NET. Results from the current study demonstrate antitumor activity in Japanese patients with unresectable, well-differentiated pancreatic NET and corroborate earlier findings in Western and Asian populations.

## Electronic supplementary material

Below is the link to the electronic supplementary material.

Table S1 (DOC 107 kb)
