# Supplementary material for: Phase II study of sunitinib in Japanese patients with unresectable or metastatic, well-differentiated pancreatic neuroendocrine tumor
Source: Invest New Drugs. 2013 Oct 1;31(5):1265–74. doi: 10.1007/s10637-012-9910-y (PMC3771378; doi:10.1007/s10637-012-9910-y)
Supplement: Supplementary file 1 [file 10637_2012_9910_MOESM1_ESM.doc]

**Supplemental Tables**

**Table S1** Gastrin levels, tumor-size changes, and overall response in patients with gastrinomas

| Patient | Time of assessment | Change from baseline in gastrin levels (%) | Change from baseline in tumor size (%) | Best overall response |
| --- | --- | --- | --- | --- |
| D | Cycle 2  (day 29) | –85 | –31 | PR |
|  | Cycle 3  (day 57) | –86 | –39 |  |
|  | Cycle 5  (day 113) | –85 | –42 |  |
|  | Cycle 7  (day 169) | –93 | –45 |  |
| L | Cycle 2  (day 30) | –9 | 15 | PD |
|  | Cycle 3  (day 58) | 17 | 30 |  |
|  | Last visit  (day 83) | 58 | – |  |

*PD* progressive disease; *PR* partial response
